# Improving Adherence to Behavioral Parent Training for ADHD Using Digital Health Tools

**DOI:** 10.20900/jpbs.20220005

**Published:** 2022-08-22

**Authors:** Linda J. Pfiffner, Melissa R. Dvorsky, Elizabeth J. Hawkey, Sara Chung, Lauren M. Haack, Elizabeth B. Owens

**Affiliations:** 1 Department of Psychiatry and Behavioral Sciences, University of California San Francisco, San Francisco, CA 94143, USA; 2 Department of Pediatrics and Psychiatry and Behavioral Sciences, Children’s National Hospital, The George Washington University School of Medicine and Health Sciences, Washington, DC 20010, USA

**Keywords:** ADHD, behavioral parent training, digital health tools

## Abstract

Behavioral Parent Training (BPT) is a well-established treatment for school-age children with ADHD but lack of parent adherence to prescribed parenting strategies limits treatment gains. Digital Health (dHealth) tools can be leveraged to target barriers to parent adherence but existing tools for parenting interventions are limited. New efforts to develop a dHealth tool to target adherence barriers including limited skill competence, EF processes, and low motivation/negative attitudes, are presented and recommendations for future technology-enhanced treatments are provided.

## IMPORTANCE OF PARENT ADHERENCE TO TREATMENT FOR ADHD

Parenting stress, parent–child conflict, and ineffective parenting are elevated in families of children with ADHD [1,2] and predict poor academic outcomes, interpersonal difficulties, and aggressive behavior [3,4]. BPT is a well-established ADHD treatment delivered in clinic settings [5] and more recently, school settings [6,7]. However, improvements in child behavior can be circumscribed and lack sustainability [8]. Poor parental adherence contributes to these limited effects [9,10], since BPT relies on parents (including primary caretakers) using recommended behavioral strategies with children on a regular basis in everyday contexts. Parents often have difficulty using prescribed strategies consistently, and further reduce their use once treatment ends [8]. Given estimates that 40%–60% of parents of children with ADHD have difficulty fully participating in treatment [8], and less than half of parents complete BPT homework assignments [11], suboptimal parent engagement and adherence are serious impediments to BPT effectiveness.

## BARRIERS TO SKILL USE

Existing studies document barriers that can impede parent adherence including: (1) *limited skill competence* (e.g., inadequate understanding or execution of skills) [12,13], (2) *interfering executive functioning(EF) processes* (e.g., forgetting to use skills; inadequate prioritizing/planning; losing materials/resources) [1,13] and (3) *low motivation/negative attitudes* (e.g., low self-efficacy, pessimism, parenting stress) [1,12,14] as well as (4) *maintenance-specific barriers* when treatment ends (e.g., lack of success adapting learned skills to new challenges; decreased accountability). Notably, ADHD and EF-related problems are common among parents of youth with ADHD [15] and related adverse effects on parent adherence may be especially pronounced at follow-up [15,16].

Surprisingly little research has focused on reducing such barriers to BPT adherence, with most efforts focused on treatment access. Some behavioral treatments have begun to address skill implementation challenges secondary to parental ADHD symptoms by providing more flexible and individualized delivery [15]. However, these adaptations can be costly, unfeasible to deliver in limited-resourced school/community mental health settings and crucially, fail to address treatment sustainability [8,15]. Feasible and practical augmentations to BPTs that address adherence barriers and skill utilization are needed to improve sustained treatment outcomes.

## ADDRESSING ADHERENCE BARRIERS WITH TECHNOLOGY

Use of technology to deliver or enhance mental health treatment has proliferated in recent years with smartphones providing on-demand and flexible access to treatment and thereby increasing treatment potency while minimizing costs [17–20]. Online portals and mobile applications that track adherence and provide feedback have demonstrated efficacy and feasibility for a variety of conditions, including for ADHD medication management [21,22].

Many digital tools specifically target parenting, but the vast majority are not empirically evaluated or based on evidence-based parenting practices [23]. Recently, however, several dHealth tools have been developed for early childhood BPTs and demonstrate promise either as augmentations [24] or alternatives to in-person treatment (“self-administered” programs) [25]. Nevertheless, existing BPT dHealth tools are limited. None have been designed for the specific needs related to parenting school-age youth with ADHD when BPT skills become more complex and children become more directly involved in treatment. Current solutions also do not specifically target adherence barriers faced by many parents of youth with ADHD who themselves are at increased risk for challenges with EF processes, low motivation, and parenting skill acquisition. Notably, use of existing technology is often less than optimal (e.g., 35%–50%+ fail to complete online modules [26,27]) and has not incorporated features to support parenting in the period following active treatment when gains often diminish [28]. Digital tools are most likely to be used if they can be incorporated into daily routines and integrated into existing face-to-face treatments and may help to address contextual issues in the environment that affect adherence. For example, digital tools may be used to help parents track personalized daily goals for themselves and their children and can prompt parents when to use specific parenting skills in real-life contexts at prescribed times (e.g., morning routine, homework time). Such prompting could potentially include a location function that delivers contextually relevant guidance depending upon location (e.g., home, car, store, restaurant). Digital tools may also provide on-demand guidance for troubleshooting problematic situations via a digital wizard/coach through analysis of relevant antecedent and consequent events. However, tools have often been designed and developed with little input from the key stakeholders [29] (parents and children with ADHD, clinicians), resulting in products that may not be engaging or useful for families or clinicians and therefore less likely to facilitate adherence or sustainability. Conceptual frameworks for behavior change can guide optimization of the delivery interface in digital behavior change interventions, but these are often not applied during development. For example, psychological theories of persuasion and attitude change, motivation, and self-regulation can inform selection and design of features [30] which may better engage purported treatment mechanisms underlying behavior change and sustainability.

## A NOVEL THEORY- AND STAKEHOLDER-INFORMED TOOL TO AUGMENT BPT

**Figure 1.**
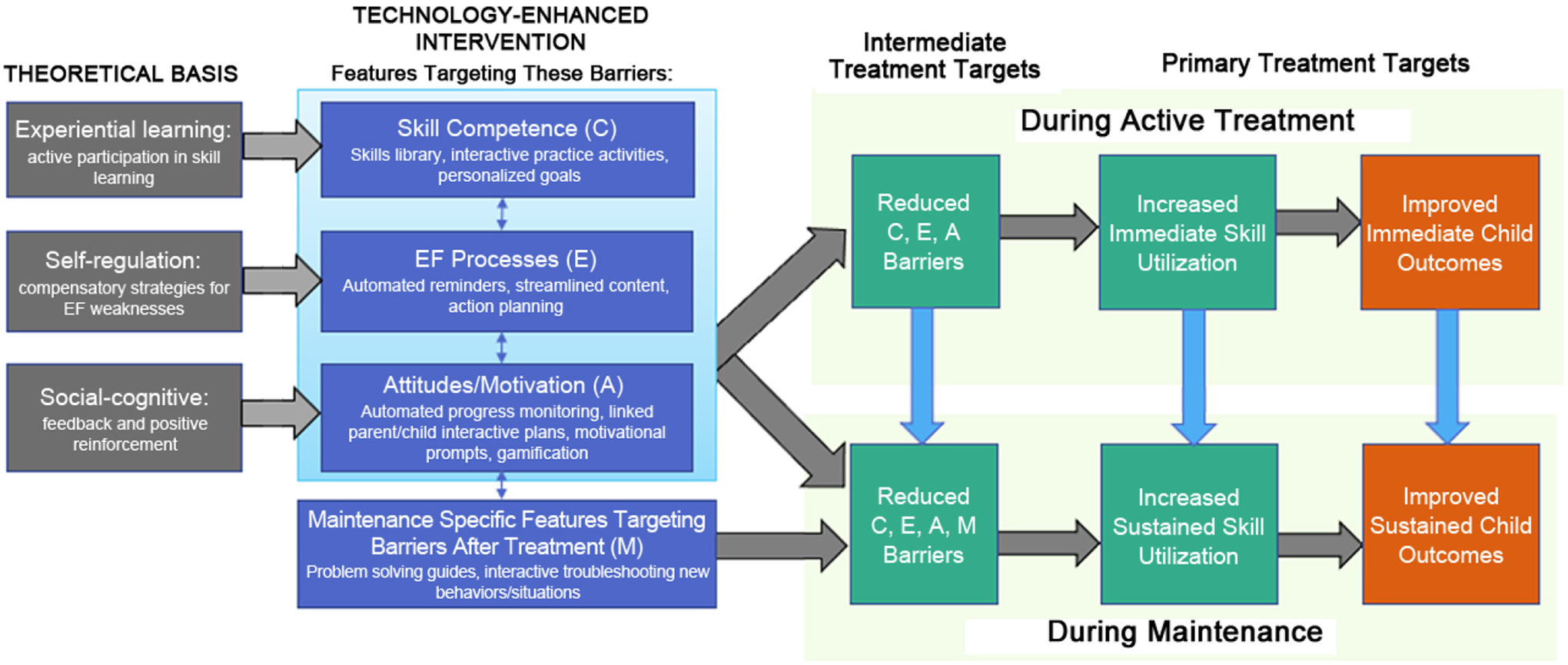
Theoretical Targets for Technology Solutions to Improve Parent Adherence and Sustained Outcomes in Behavior Parent Training for Youth with ADHD.

With support from the National Institute of Mental Health (R34 MH122222), our team is designing a dHealth tool to augment school-based BPT for ADHD that addresses the limitations above. Key stakeholders (parents, children, school clinicians) are informing each iteration of tool development in the context of a user-centered, mixed method design. As depicted in our conceptual model (Figure 1), the tool targets empirically-supported barriers to parent adherence [12,13]: skill competence, EF processes, and motivation/attitudes. We applied frameworks for digital behavior change interventions [30–32], to select the primary features addressing each barrier. For example, features addressing *Skill Competence* were informed by experiential learning theory [32] and include interactive practice activities via skill modules which provide opportunities for active learning and engage parents in setting their own personalized short-term goals and action planning [20,33]. Features for overcoming barriers due to *Executive Functioning Processes* are informed by self-regulation theories [30] and include automated reminders, streamlined content, and personalized goal-setting and action planning. Features to enhance *Motivation and Attitudinal Processes* for skill utilization [34] are guided by social cognitive theory [30] and include personalized goal-setting, automated monitoring of parent/child progress, motivational prompts, and gamification (digital rewards for skill use). Linked parent/child views of child goals and reward plans integrated in daily routines address EF and motivational barriers since children who earn rewards when parents check their behavior goals are likely to remind and motivate parents to use the tool. To support sustained and independent parental skill use, *Maintenance Specific Features* include navigation for personalized problem-solving guides parents and troubleshooting new behavior problems by prompting learned skills. As depicted Figure 1, reductions in these targeted barriers are hypothesized to increase immediate and sustained parent skill utilization which in turn is expected to improve immediate and sustained child outcomes.

## IMPLICATIONS FOR FUTURE RESEARCH

DHealth tools are promising, but have yet to be fully realized as effective methods for addressing adherence problems and sustaining treatment effects. Key elements likely to optimize effects of digital augmentations include: (1) developing tools specifically targeting empirically-supported barriers to adherence based on the intervention theory of change, (2) incorporating conceptually relevant and empirically-supported features, and (3) using an iterative user-centered design with families affected by the target condition and their clinicians to ensure usability and utility of the tool. The potential for broad uptake and scalability of digital interventions has never been greater. By including theory-informed features, targeting key treatment mechanisms, and involving stakeholders throughout the development process, current efforts leveraging technology will be better positioned to increase efficacy and reach of evidence-based practices.
